# Metacognitive Training Modulates Default-Mode Network Homogeneity During 8-Week Olanzapine Treatment in Patients With Schizophrenia

**DOI:** 10.3389/fpsyt.2020.00234

**Published:** 2020-03-27

**Authors:** Xiaoxiao Shan, Rongyuan Liao, Yangpan Ou, Yudan Ding, Feng Liu, Jindong Chen, Jingping Zhao, Wenbin Guo, Yiqun He

**Affiliations:** ^1^Department of Psychiatry, The Second Xiangya Hospital of Central South University, Changsha, China; ^2^National Clinical Research Center on Mental Disorders, Changsha, China; ^3^The Second Affiliated Hospital of Xinxiang Medical University, Xinxiang, China; ^4^Department of Radiology, Tianjin Medical University General Hospital, Tianjin, China

**Keywords:** metacognitive training, default-mode network, network homogeneity, schizophrenia, olanzapine

## Abstract

**Background:**

Previous studies have revealed the efficacy of metacognitive training for schizophrenia. However, the underlying mechanisms of metacognitive training on brain function alterations, including the default-mode network (DMN), remain unknown. The present study explored treatment effects of metacognitive training on functional connectivity of the brain regions in the DMN.

**Methods:**

Forty-one patients with schizophrenia and 20 healthy controls were scanned using resting-state functional magnetic resonance imaging. Patients were randomly assigned to drug plus psychotherapy (DPP) and drug therapy (DT) groups. The DPP group received olanzapine and metacognitive training, and the DT group received only olanzapine for 8 weeks. Network homogeneity (NH) was applied to analyze the imaging data, and pattern classification techniques were applied to test whether abnormal NH deficits at baseline might be used to discriminate patients from healthy controls. Abnormal NH in predicting treatment response was also examined in each patient group.

**Results:**

Compared with healthy controls, patients at baseline showed decreased NH in the bilateral ventral medial prefrontal cortex (MPFC), right posterior cingulate cortex (PCC)/precuneus, and bilateral precuneus and increased NH in the right cerebellum Crus II and bilateral superior MPFC. NH values in the right PCC/precuneus increased in the DPP group after 8 weeks of treatment, whereas no substantial difference in NH value was observed in the DT group. Support vector machine analyses showed that the accuracy, sensitivity, and specificity for distinguishing patients from healthy controls were more than 0.7 in the NH values of the right PCC/precuneus, bilateral ventral MPFC, bilateral superior MPFC, and bilateral precuneus regions. Support vector regression analyses showed that high NH levels at baseline in the bilateral superior MPFC could predict symptomatic improvement of positive and negative syndrome scale (PANSS) after 8 weeks of DPP treatment. No correlations were found between alterations in the NH values and changes in the PANSS scores/cognition parameters in the patients.

**Conclusion:**

This study provides evidence that metacognitive training is related to the modulation of DMN homogeneity in schizophrenia.

## Introduction

Schizophrenia is a chronic disorder with a high functional disability. Previous studies have shown that approximately 20% to 30% of patients with schizophrenia are resistant to antipsychotics (1). Compliance with medication remains low, even during atypical antipsychotic medication (2, 3). Hence, therapy based on pharmacological treatment may not attain satisfactory improvement of social functioning in the patients (4).

Metacognition is an extensive mental activity that involves contemplation of one’s own thinking or others’ mental state. Metacognitive deﬁcits have been reported in all phases of schizophrenia (5–7) and are related to poor treatment outcomes. Metacognitive training (MCT), a novel and widely used group intervention for patients with schizophrenia, may enhance patients’ self-awareness and insights into these cognitive distortions to alleviate the positive symptoms of psychosis, especially paranoid ideation(8). A systematic review that covered 14 studies showed that MCT effectively decreased cognitive biases and delusions related to schizophrenia and improved insight in the patients with schizophrenia (9). Randomized controlled trials showed that MCT reduced positive symptomatology and influenced insight and social functioning (10, 11). Moritz et al. found that MCT exhibited sustained effects in the decrease of delusions, which were beyond the effects of antipsychotic drugs; meanwhile, the quality of life and self-esteem in the MCT group had a significant improvement after 3 years of treatment (12). However, the potential mechanisms through which MCT executes treatment effects remain unknown.

As the major components of the default-mode network (DMN), medial prefrontal cortex (MPFC), posterior cingulate cortex (PCC), and precuneus play a key role in metacognition capacity and related cognitive functions, including self-referential tasks (13, 14). The eﬃcient connectivity within the DMN is crucial for high metacognitive capacity (15). Francis et al. discovered substantial resting-state functional connectivity across the precuneus, MPFC, and PCC in patients with schizophrenia with high metacognitive capacity; this finding suggests that disrupted resting-state connectivity is relevant to metacognitive dysfunction in psychosis (15). Baird et al. determined relationships between resting-state functional connectivity in the precuneus, anterior MPFC, and inferior parietal lobule structures/intraparietal sulcus and metacognitive capacity relevant to memory retrieval (16). However, it remains unknown whether MCT can modulate functional connectivity of the DMN.

In the present study, alterations in the DMN homogeneity relative to MCT treatment in patients with schizophrenia were examined using network homogeneity (NH) (17) from a network-based perspective. NH is defined as the mean associations between the time series of a given voxel and the time series of all other voxels inside the network. NH allows an unbiased examination to a network of interest by seeking for brain regions that reveal pathology correlated with alterations in this network. Many clinical studies on psychiatric disorders have used NH, particularly in patients with schizophrenia and their unaffected siblings (18, 19), major depressive disorders (20), attention deficit/hyperactivity disorder (17), and somatization disorder (21). We examined DMN alterations at two time points (baseline and 8 weeks of treatment) in inpatients with schizophrenia at rest. We hypothesized that MCT could modulate DMN homogeneity in patients with schizophrenia, particularly in the MPFC, PCC, and precuneus. Correlations between alterations in the DMN NH values and reductions in symptom severity were also expected in this study. We also hypothesized that the abnormalities of DMN NH at baseline might be applied as underlying image biomarkers for distinguishing patients from controls through the support vector machine (SVM) analyses.

## Materials and Methods

### Participants

We recruited forty-one patients with schizophrenia from the Second Affiliated Hospital of Xinxiang Medical University in China. Twenty patients were allocated to the drug plus psychotherapy (DPP) group, and twenty-one patients were allocated to the drug therapy (DT) group on the basis of the random number list. The assessment was executed by a psychiatrist who was blinded to the patient allocation. Schizophrenia was diagnosed using the Structural Clinical Interview for Diagnostic and Statistical Manual of Mental Disorders, Fifth Edition (DSM-5). Illness duration was no more than five years since the onset of the disease, and the total score of the positive and negative syndrome scale (PANSS) was greater than 75. The patients were right-handed and 18–50 years old. They were randomly allocated to the DPP and DT groups. PANSS was applied to assess symptomatic severity at baseline and 8 weeks of treatment. Cognitive function was evaluated through the Measurement and Treatment Research to Improve Cognition in Schizophrenia Consensus Cognitive Battery, including Trail-making test, part A (TMT-A); Hopkins verbal learning Test–Revised (HVLT-R); Brief Assessment of Cognition in Schizophrenia Symbol Coding Test (BACS-SC); Brief Visuospatial Memory Test–Revised (BVMT-R); Continuous Performance Test-identical Pairs (CPT-IP); Wechsler Memory Scale Spatial Span (WMS-SS); Neuropsychological Assessment Battery–Mazes(NAB-M); Mayer–Salovey–Caruso Emotional Intelligence Test (MSCEIT); and Category Fluency–Animal Naming Fluency (CF–ANF). These tests were used to evaluate processing speed, attention/vigilance, working memory, verbal learning, reasoning, and problem solving in the participants.

Healthy controls unrelated to patients were recruited from the local community. Age, years of education, and sex ratio of the patients and healthy controls were matched. The non-patient version of the Structured Clinical Interview for DSM-IV was used to screen healthy controls. Healthy controls were excluded if they suffered from any medical and neurological illnesses, substance abuse, and psychosis symptoms. They were also excluded if they had a first-degree relative with a history of psychiatric disorders.

The exclusion criteria for all individuals were as follows: any current or past neuropsychiatric disorders; any physical illnesses, such as cardiovascular and liver and kidney diseases; any traumatic brain injury; serious impulsive behavior; seizures; a history of electroconvulsive therapy and olanzapine therapy that were ineffective or tolerable; drug or alcohol addiction; pregnancy; and contraindications for MRI scan.

The study was approved by the local ethics committee of the Second Affiliated Hospital of Xinxiang Medical University, and has been registered in a public trials registry. The number is NCT03451734 from ClinicalTrials.gov. The study was executed in accordance with the Helsinki Declaration. After a complete explanation, the participants provided their written informed consent.

### Intervention

Olanzapine with a mean dose of 21.58 and 20.50 mg/day was administered to each patient in the DPP and DT groups, respectively. Olanzapine dosage increased within the first 2 weeks and remained unchanged until the last fMRI scan. The use of other antipsychotic drugs was not allowed. On the basis of the olanzapine treatment, the DPP group received MCT from a trained clinical psychologist who had more than one years of experience with MCT. The DT group was given a non-specific therapeutic procedure involving some recreational activities as executed in the Second Affiliated Hospital of Xinxiang Medical University. The duration of entire program and frequency and duration of the sessions were matched to the MCT program.

MCT consisted of eight sessions. The details of MCT procedure are offered in the **Supplementary Method**.

### Image Acquisition and Processing

A 3.0 T Siemens scanner (Germany) was used to scan the subjects. All participants were instructed to lie on the scanner with their eyes closed and stay still. Soft earplugs and foam pads were used to reduce scanner noise and head motion. The parameters were as follows: flip angle = 90°, repetition time/echo time = 2000/30 ms, slice thickness/inter-slice spacing = 4/0.8 mm, field of view = 220 mm × 220 mm, and acquisition matrix = 64×64. Each resting-state fMRI scan contained 240 image volumes.

The resting-state fMRI data were preprocessed by the DPABI software (22). The ﬁrst 10 volumes were ruled out because of the instability of the initial MRI signal and for the individuals to adapt to circumstances. Subjects with over 2-mm maximal translation in the x, y, or z axis and 2° maximal rotation in each axis were excluded after slice timing and head motion correction. The imaging data were then spatially normalized to a conventional Montreal Neurological Institute (MNI) EPI template and resampled to 3 × 3 × 3 mm. The follow-up images of the patients were coregistered with baseline images before normalization. Finally, the data were temporally band-pass filtered (0.01–0.08 Hz) and linearly detrended to reduce the effect of physiological high-frequency noise and low-frequency drifts. Several covariates, including signals from a ventricular region of interest, signals from a region centered in the white matter, and Friston-24 head motion parameters obtained *via* rigid body correction, were removed. The global signal was not removed as indicated in a previous study (23). Besides, mean framewise displacement (FD) was used to solve the residual effects of motion as a covariate in group analyses. Scrubbing was also used as an aggressive head motion control strategy (removing time points with FD > 0.2mm) to minimize confounding effects of head motion.

### DMN Identification

After being preprocessed, the time series of all the groups were examined to construct a DMN mask by using Group ICA with the toolbox GIFT (http://mialab.mrn.org/software/#gica) (24, 25). The details of DMN identification are offered in the **Supplementary Method**.

### NH Analyses

NH analyses were performed with an in-house MATLAB script. The details of NH analyses are offered in the **Supplementary Method**.

### Statistical Analyses

Data about the demographic characteristics of the three groups were compared by using the Mann–Whitney U-test or the chi-square test when necessary.

Repeated analyses of covariance (ANCOVAs) were conducted to analyze the differences of the three groups on the NH maps, and to assess the interaction effects between time points and groups with age and mean framewise displacement as the covariates. Post hoc t-tests were used to evaluate group differences. Gaussian random field (GRF) theory was used to correct for multiple comparisons at p < 0.05 with the REST software (voxel significance: p < 0.001, cluster significance: p < 0.05).

To assess the treatment effect, the following formula was used to calculate the reduction ratio (RR) of the PANSS total scores.

$$\mathrm{RR}=\left({\mathrm{PANSS}}_{\mathrm{total}\_1}-{\mathrm{PANSS}}_{\mathrm{total}\_2}\right)/{\mathrm{PANSS}}_{\mathrm{total}\_1}$$

PANSS_total_1_ referred to the PANSS total scores at baseline, whereas PANSS_total_2_ was the PANSS total scores after 8 weeks of treatment. Similar RRs were calculated for the PANSS positive, negative, and general symptoms subscale scores.

### Correlation Analyses

After abnormal NH of the brain clusters were identified, the average NH values from these clusters were extracted. The correlations between abnormal NH and PANSS scores/cognition parameters of the patients at baseline and between NH alterations and changes in PANSS scores/cognition parameters of the patients after treatment were determined using Pearson’s correlation analyses with a threshold of p < 0.05.

### Classification Analysis Using SVM

SVM was applied to test the capability of abnormal NH values in any brain region to distinguish patients from healthy controls by using the LIBSVM software package (http://www.csie.ntu.edu.tw/~cjlin/libsvm/) in MATLAB. The “leave one out” method was used.

Support vector machines (SVM), a popular and well-known supervised learning technique, is used to find a max-margin separator hyperplane to classify data (26). The hyperplane is orientated that it is as far as possible from the nearest data points from each class. These nearest points are called the support vectors (27).

Given a labeled training dataset:

$$(x_{1},\;y_{1}),\ldots ,\text{ (}x_{n},\;y_{n}),\;x_{i}\in R^{d}\;\mathrm{and}\;y_{i}\in (-1,\;+1),$$

where X_i_ ∈ R^d^ are the training data and Y_i_ ∈ {−1, 1} are the corresponding labels. The essence of SVM is to find the optimal hyperplane and to divide the two classes of data points with the maximum margin (28):

(1) $$\begin{array}{c} \min\\ \mathrm{wb} \end{array}\;\frac{1}{2}\|\;w\|2+C\sum_{i=1}^{n}\xi i$$

$$s.\;t.\;Y_{i}\left(X_{i}w+\;b\right)\;\geq \;1\;–\;\xi_{i}$$

$$\xi_{i}\geq \;0,\;i\;=\;0,\;1,\;2,\ldots ,\;n.$$

Here, C is a tuneable positive scalar and ξ_i_ is the slack variables. It may be equivalently converted into hinge loss with an ℓ2 norm penalty format:

(2) $$\begin{array}{c} \min\\ \mathrm{wb} \end{array}\;\Sigma_{i=1}^{n}\;{\left(1\;-\;Y_{i}(X_{i}w+b)\right)}_{+}+\frac{\lambda^{\prime }}{2}{\left||w|\right|}^{2}$$

where the loss function (1 − ·)_+_ = max(1 − ·, 0) is called hinge loss and λ′ is positive regularization parameter corresponding to C parameter in problem (1), which helps control the balance between the loss and penalty. A more detailed description about SVM can be found in a previous study (29).

### Classification Analysis by Using SVR

Support vector regression (SVR) was used to explore the capability of the extracted NH values in abnormal brain regions to predict treatment response with the LIBSVM software package (http://www.csie.ntu.edu.tw/~cjlin/libsvm/) in MATLAB. SVR was executed for the extracted baseline levels of NH values and each symptomatic domain (PANSS total, positive symptoms, negative symptoms, and general symptom subscale scores) in each patient group.

Detecting a multivariate regression function f(x) based on X through a sample spectrum is to predict a desired output feature. The SVR equation has been clearly clarified in the literature (30, 31).

## Results

### Demographic and Clinical Characteristics

**Figure 1:**
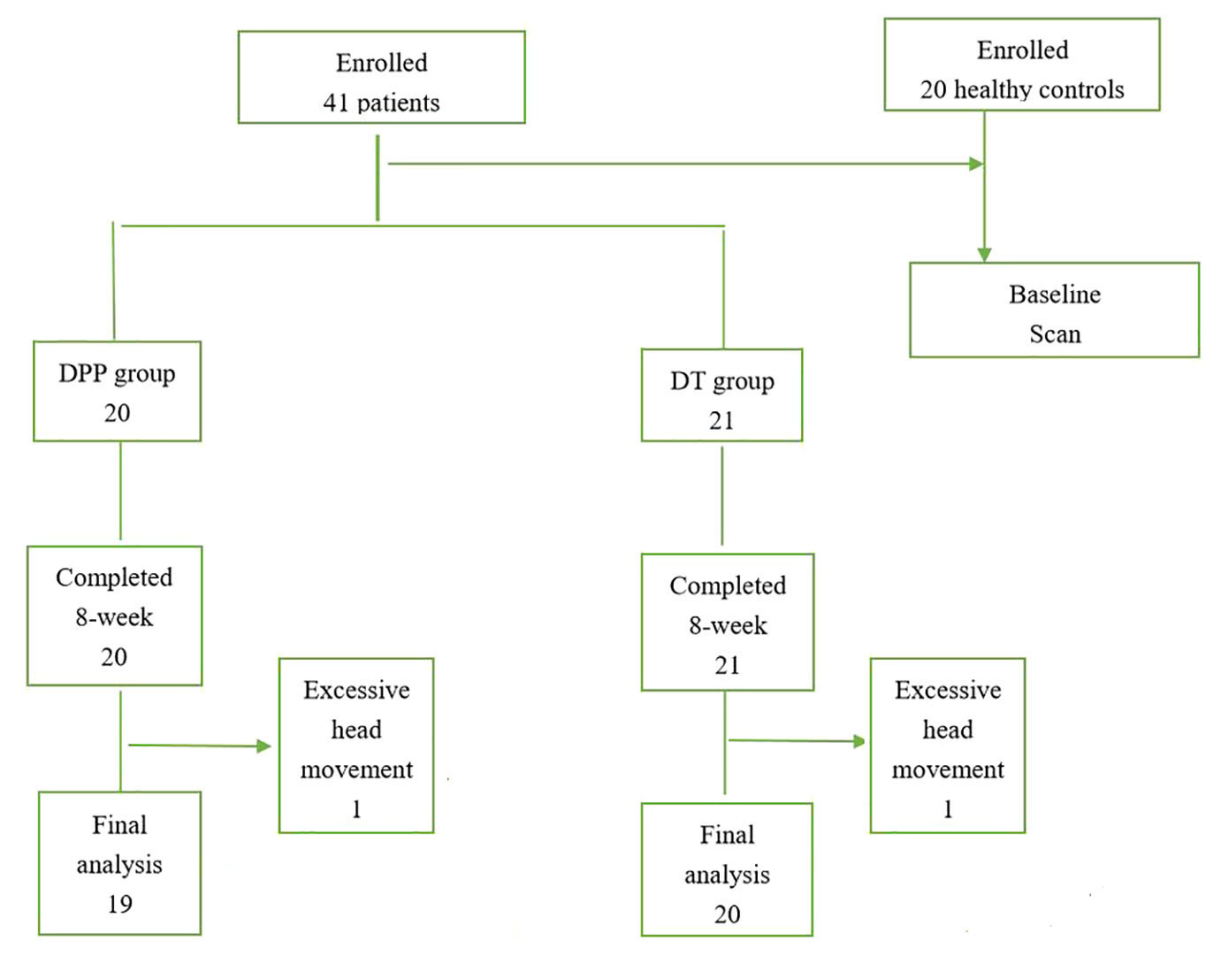
The consort flow diagram for the present study.

A total of 41 patients with schizophrenia and 20 healthy controls were enrolled in the study. However, two patients (one in the DPP group and one in the DT group) were excluded due to excessive head movement. A total of 39 patients with schizophrenia (19 in the DPP group and 20 in the DT group) were included in the analysis (**Figure 1**). No substantial differences were observed in the age, sex ratio, and years of education across the three groups. The mean dosage of olanzapine did not significantly differ between the DPP and DT groups (**Table 1**). No signiﬁcant differences were found in the negative and general symptoms subscale scores of PANSS between the two groups, except the positive symptoms subscale score (p < 0.05) at baseline.

**Table 1:** Demographic characteristics of the subjects.

	DPP(*n* = 19)	DT (*n* = 20)	Controls (*n* = 20)	F/χ^2^	*p*
**Sex (male/female)**	12/7	15/5	14/6	0.648	0.723^a^
**Age (years)**	26.05 ± 5.81	22.75 ± 4.38	25.70 ± 4.90	5.667	0.059^b^
**Years of education (years)**	11.63 ± 3.75	10.65 ± 2.50	12.75 ± 2.95	4.120	0.127^b^
**Dose of olanzapine (mg/day)**	21.58 ± 3.75	20.50 ± 1.54		0.459	0.498^b^

a The *p* values for sex distribution were obtained by a chi-square test.

b The *p* values were obtained by Mann-Whitney *U* test.

DPP, drug plus psychotherapy; DT, drug therapy.

### Clinical Symptoms After 8 Weeks of Treatment

**Figure 2:**
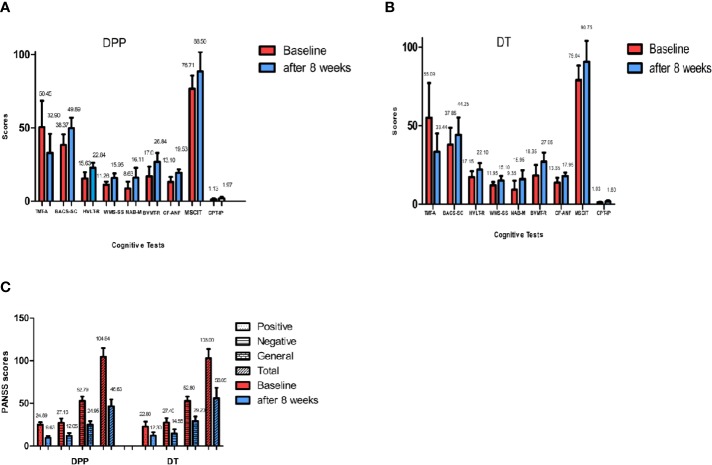
PANSS and cognitive tests results across different time points. Values above histogram bars represent related group means. Bars represent related SD. PANSS, Positive and Negative Syndrome Scale; TMT-A, Trail Making Test, part A; BACS-SC, Brief Assessment of Cognition in Schizophrenia Symbol Coding Test; HVLT-R, Hopkins Verbal Learning Test-Revised; WMS-SS, Wechsler Memory Scale Spatial Span; NAB-M, Neuropsychological Assessment Battery-Mazes; BVMT-R, Brief Visuospatial Memory Test-Revised; CF-ANF, Category Fluency-Animal Naming Fluency; MSCIT, Mayer-Salovey-Caruso Emotional Intelligence Test; CPT-IP, Continuous Performance Test-identical Pairs. **(A)** Cognitive results across different time points in the DPP group. **(B)** Cognitive results across different time points in the DT group. **(C)** PANSS total scores and subscale scores across different time points in the two groups.

There are significant interactions of group × time for PANSS total scores, positive, and general symptoms subscale scores. By contrast, the interaction of group × time was not significant for PANSS negative symptoms subscale scores. DPP group and DT group showed significant improvement in PANSS total scores, positive symptoms subscale scores, negative symptoms subscale scores, general symptoms subscale scores, and cognitive function tests after 8 weeks of treatment compared to the baseline values (**Figure 2**) (p ≤ 0.001). After 8 weeks of treatment, the PANSS total, positive symptoms, and general symptoms subscale scores in the DPP group became considerably lower than those in the DT group (46.64 ± 7.97 VS 56.05 ± 12.08;9.63 ± 2.24 VS 12.3 ± 3.85; 24.95 ± 4.08 VS 29.2 ± 5.51, respectively) (p < 0.05). The CF-ANF and BACS-SC scores in the DT group were also substantially lower than those in the DPP group (17.95 ± 2.26 vs 19.53 ± 2.25; 44.25 ± 11.02 vs 49.89 ± 7.10, respectively) (p < 0.05) (**Table 2**). Besides, the BACS-SC, HVLT-R, WMS-SS, CF-ANF scores showed interactions of group × time.

**Table 2:** Comparison of the clinical characteristics between the DPP group and the DT group.

			Baseline		Z	P	Z^a^	8 weeks		Z	P	Z^b^
	Test Statistic	P	DPP group	DT group				DPP group	DT group			
PANSS Time Group × Time	F= 829.876 F= 9.515	0.000 0.0038	104.84 ± 9.96	103.0 ± 10.79	-0.394	0.708	-3.898	46.64 ± 7.97	56.05 ± 12.08	-2.169	0.03	-3.922
Positive Time Group × Time	F=281.287 F=9.615	0.000 0.0037	24.89 ± 3.09	22.80 ± 5.82	-2.003	0.047	-3.826	9.63 ± 2.24	12.3 ± 3.85	-2.426	0.015	-3.886
Negative Time Group × Time	F= 221.783 F= 1.443	0.000 0.237	27.16 ± 5.19	27.4 ± 5.42	0.000	1.0	-3.825	12.05 ± 3.19	14.55 ± 5.12	-1.27	0.241	-3.929
General Time Group × Time	F= 980.166 F= 6.665	0.000 0.014	52.79 ± 5.13	52.8 ± 5.11	-0.014	0.989	-3.828	24.95 ± 4.08	29.2 ± 5.51	-2.439	0.014	-3.921
TMT-A Time Group × Time	F=68.759 F=0.751	0.000 0.392	50.45 ± 18.13	55.09 ± 22.11	-0.72	0.496	-3.823	32.90 ± 13.08	33.44 ± 11.54	-0.267	0.792	-3.92
BACS-SC Time Group × Time	F=78.836 F=6.447	0.000 0.015	38.37 ± 7.28	37.85 ± 10.79	-1.17	0.247	-3.729	49.89 ± 7.10	44.25 ± 11.02	-2.293	0.021	-3.929
HVLT-R Time Group × Time	F=173.030 F=5.979	0.000 0.019	15.63 ± 4.19	17.15 ± 3.79	-1.03	0.309	-3.831	22.84 ± 3.39	22.1 ± 4.09	-0.339	0.749	-3.84
WMS-SS Time Group × Time	F=134.380 F=5.154	0.000 0.029	11.26 ± 2.1	11.95 ± 2.65	-0.575	0.588	-3.832	15.95 ± 3.08	15.1 ± 2.94	-1.018	0.322	-3.947
NAB-M Time Group × Time	F=95.522 F=0.368	0.000 0.548	8.63 ± 4.78	9.35 ± 5.66	-0.042	0.967	-3.828	16.11 ± 6.67	15.95 ± 5.71	-0.07	0.945	-3.929
BVMT-R Time Group × Time	F=127.686 F=0.484	0.000 0.491	17.0 ± 6.68	18.35 ± 6.62	-0.451	0.667	-3.828	26.84 ± 6.06	27.05 ± 5.81	-0.183	0.857	-3.924
CF-ANF Time Group × Time	F=214.891 F=8.411	0.000 0.006	13.10 ± 3.40	13.65 ± 3,22	-0.82	0.428	-3.833	19.53 ± 2.25	17.95 ± 2.26	-2.163	0.033	-3.932
MSCIT Time Group × Time	F=69.445 F=0.001	0.000 0.979	76.71 ± 9.01	79.04 ± 9.19	-0.843	0.411	-3.823	88.50 ± 13.25	90.75 ± 13.28	-0.702	0.496	-3.92
CPT-IP Time Group × Time	F=72.904 F=0.167	0.000 0.685	1.13 ± 0.89	1.03 ± 0.56	-0.098	0.923	-3.823	1.97 ± 0.97	1.80 ± 0.54	-0.759	0.461	-3.92

Z: The comparison between the DPP group and the DT group using Wilcoxon test of two independent samples.

Z^a^: The comparison from baseline to 8weeks within DPP group using Pairs Sample Wilcoxon test.

Z^b^: The comparison from baseline to 8weeks within DT group using Pairs Sample Wilcoxon test.

TMT-A, Trail Making Test, part A; BACS-SC, Brief Assessment of Cognition in Schizophrenia Symbol Coding Test; HVLT-R, Hopkins Verbal Learning Test-Revised; WMS-SS, Wechsler Memory Scale Spatial Span; NAB-M, Neuropsychological Assessment Battery-Mazes; BVMT-R, Brief Visuospatial Memory Test-Revised;CF-ANF, Category Fluency-Animal Naming Fluency; MSCIT, Mayer-Salovey-Caruso Emotional Intelligence Test; CPT-IP, Continuous Performance Test-identical Pairs.

### DMN Mask

The group ICA approach was applied to generate a DMN mask for all subjects. The DMN included MPFC, precuneus, PCC, lateral parietal and temporal gyri, and cerebellum Crus II. The obtained DMN mask was used in the following NH analysis.

### Repeated ANCOVA Results

**Figure 3:**
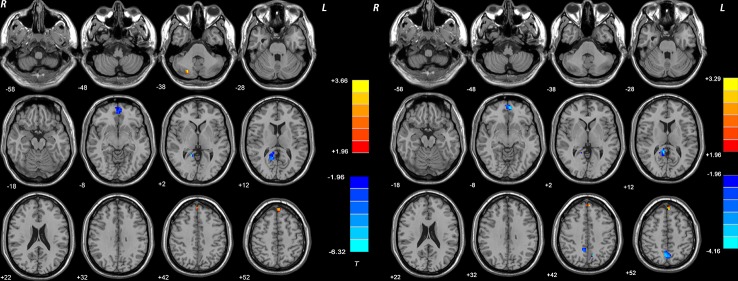
Brain regions with significant difference in DMN NH between patients and healthy controls at baseline. The color bar represents the t values of the group analysis of NH. Left: DPP group vs healthy controls. Brain regions with significant difference were observed in the right Cerebellum Crus II, bilateral ventral MPFC, right Precuneus/PCC, and bilateral superior MPFC. Right: DT group vs healthy controls. Brain regions with significant difference were observed in the bilateral ventral MPFC, right Precuneus/PCC, bilateral Precuneus, and bilateral superior MPFC. DPP, drug plus psychotherapy; DT, drug therapy; DMN, default-mode network; NH, network homogeneity; MPFC, medial prefrontal cortex; PCC, posterior cingulate cortex.

**Figure 4:**
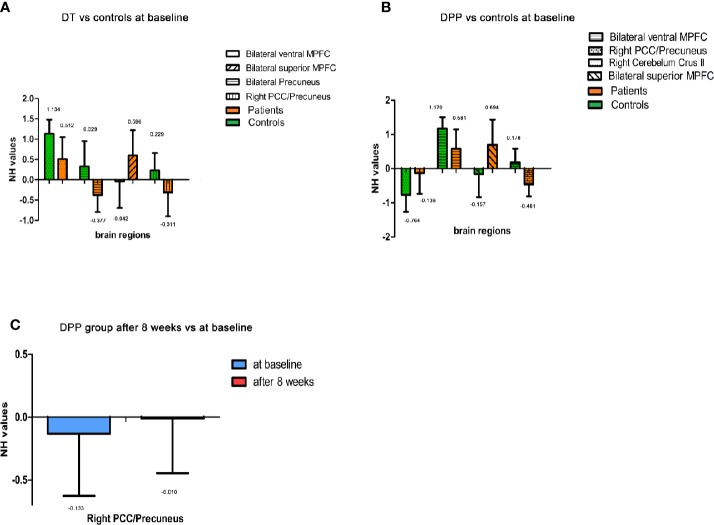
Imaging results across different time points. Values above histogram bars represent related group means. Bars represent related SD. **(A)** The NH values of brain regions across patients in the DT group and controls at baseline. **(B)** The NH values of brain regions across patients in the DPP group and controls at baseline. **(C)** The NH values of brain regions in the DPP group from baseline to 8 weeks.

**Figure 5:**
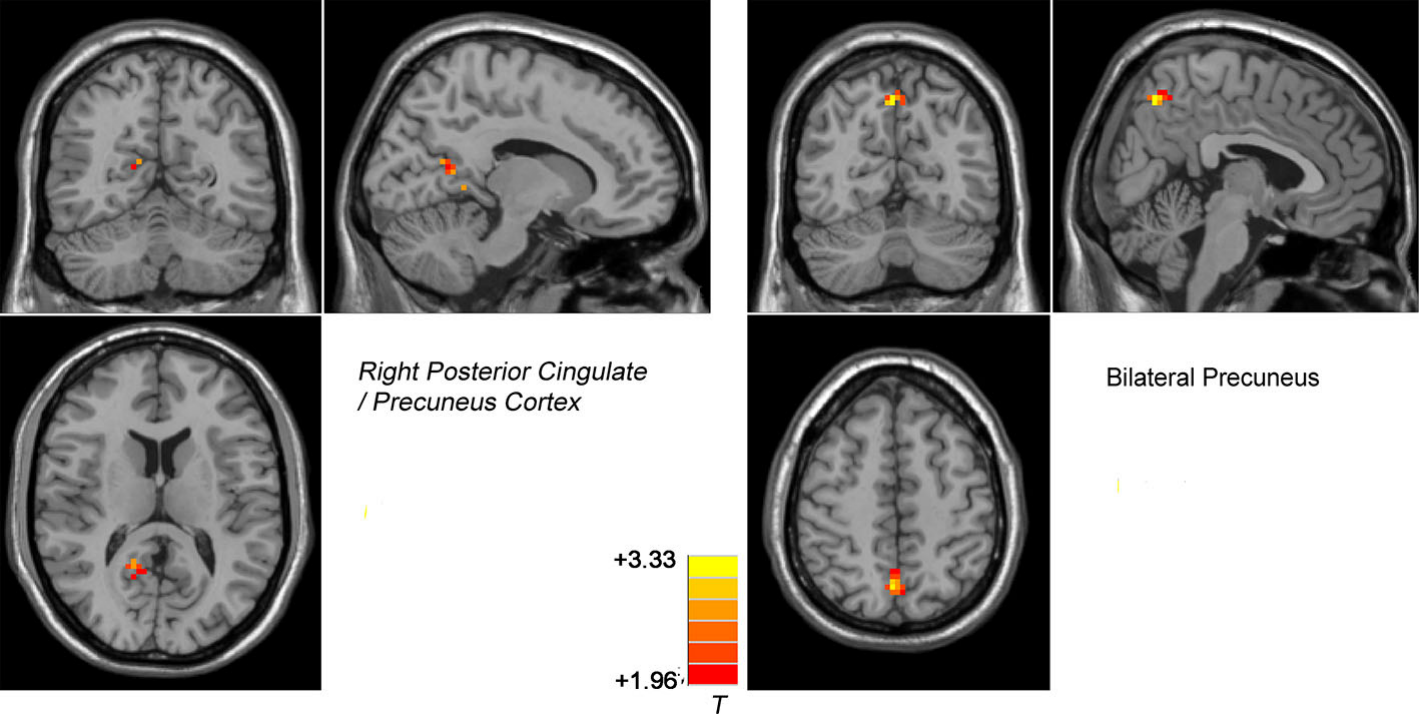
Treatment effects of DMN NH across patients at two point (at baseline and after 8 weeks of treatment). The color bar represents the t value of the group analysis of NH. Left: DPP group from baseline to 8 weeks. Brain regions with significant difference in the NH values were observed in the right Precuneus/PCC. Right: DT group vs DPP group at 8 weeks. Brain regions with significant difference in the NH value were observed in the bilateral Precuneus.

Compared with the controls, the patients (both DPP and DT groups) at baseline showed decreased NH in the bilateral ventral MPFC, right PCC/precuneus, and bilateral precuneus and increased NH in the right cerebellum Crus II and bilateral superior MPFC (**Table 3** and **Figures 3** and **4**). Compared with the baseline parameters, the DPP group showed increased NH in the right PCC/precuneus after treatment (**Table 3**, **Figures 4** and **5**), whereas the DT group showed no remarkable difference in the NH values after 8 weeks of treatment. After treatment, patients in the DPP group showed increased NH in the bilateral precuneus compared with those in the DT group (**Table 3**, **Figure 5**). At baseline, no signiﬁcant difference in the NH values was found between the two groups. Besides, the significant group × time interactions on NH are presented in the **Supplemental Figures** and **Tables**.

**Table 3:** Alterations of DMN NH across patients (at baseline, after 8 weeks of treatment) and controls.

Cluster location	Peak coordinate			Cluster (voxel)	*T* value
	x	y	Z		
**DPP group at baseline vs controls**					
Right Cerebellum Crus II	24	-75	-39	20	3.661
Bilateral ventral MPFC	0	54	-6	58	-3.3422
Right PCC/Precuneus	15	-48	3	65	-6.3186
Bilateral superior MPFC	-3	48	54	26	3.4227
**DT group at baseline vs controls**					
Bilateral ventral MPFC	-3	54	-9	53	-3.8297
Right PCC/Precuneus	9	-45	9	38	-4.1595
Bilateral Precuneus	3	-63	51	75	-3.7244
Bilateral superior MPFC	-3	42	51	23	3.1693
**DPP group after 8 weeks vs at baseline**					
Right PCC/Precuneus	15	-48	6	21	3.1264
**DT group after 8 weeks vs at baseline**					
No cluster					
**DPP group vs DT group after 8 weeks**					
Bilateral Precuneus	3	-66	51	24	3.3257
**DPP group vs DT group at baseline**					
No cluster					

The significance level was set at p < 0.05 corrected by the Gaussian random field (GRF) theory (voxel significance: p < 0.001, cluster significance: p < 0.05) for multiple comparisons with the REST software (age and mean FD as covariates). MPFC, medial prefrontal cortex; PCC, posterior cingulate cortex; FD, framewise displacement.

### Correlation Results

**Figure 6:**
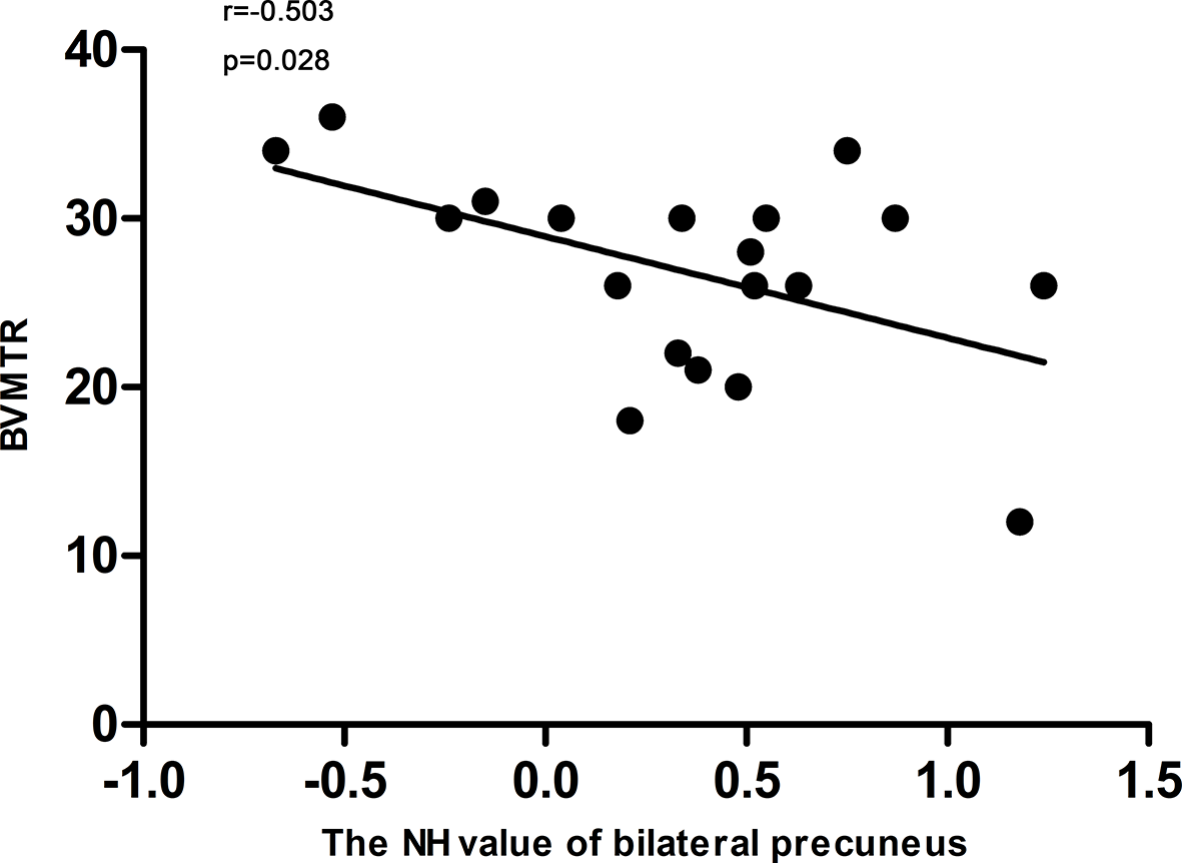
Correlations between the NH values of the bilateral Precuneus and the BVMTR scores in patients after 8 weeks of treatment.

The NH value of the bilateral precuneus, which was different in this brain regions between DPP group and DT group after 8 weeks of treatment, was negatively correlated with BVMTR in the DPP group (r=-0.503, p=0.028) (**Figure 6**). There were no signiﬁcant correlations between alterations in the NH values and changes in the PANSS scores/cognition parameters in the DPP group and DT group, respectively, and no significant correlation was observed between abnormal NH and symptomatic severity in the DPP group and DT group, respectively.

### Discriminating Patients From Controls

**Table 4** presents the detailed information of the SVM results for DPP and DT groups.

**Table 4:** Discriminating patients from healthy controls by the SVM analyses.

Brain region	Accuracy	Sensitivity	Specificity
DPP group			
Right PCC/Precuneus	89.74%	84.21%	95.00%
Right Cerebellum Crus II	74.36%	68.42%	80.00%
Bilateral ventral MPFC	79.49%	73.68%	85.00%
Bilateral superior MPFC	79.49%	68.42%	90.00%
DT group			
Bilateral ventral MPFC	80.00%	90.00%	70.00%
Right PCC/Precuneus	80.00%	70.00%	90.00%
Bilateral Precuneus	77.50%	80.00%	75.00%
Bilateral superior MPFC	72.50%	75.00%	70.00%

PCC, posterior cingulate cortex; SVM, support vector machine.

**Figure 7:**
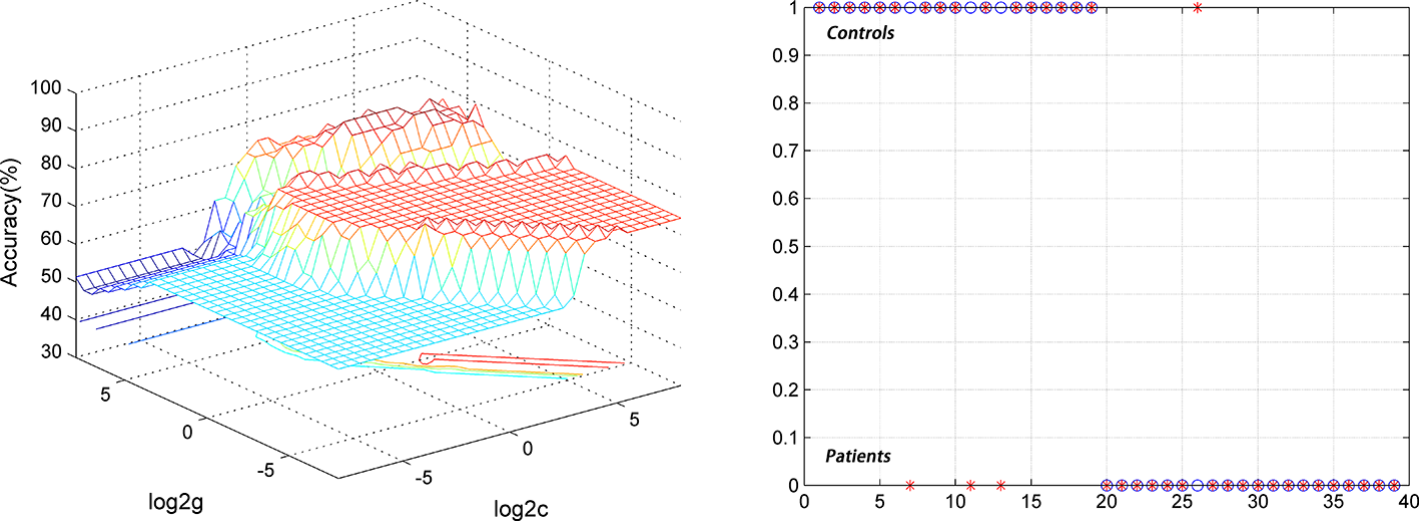
Using decreased NH values in the right PCC/precuneus to differentiate the patients (DPP group) from the controls. Visualization of classifications through support vector machine (SVM) using the NH values in the significantly different regions. Left: SVM parameters selection result of 3D view; Right: Classiﬁed map of the NH values in the right PCC/precuneus. SVM, support vector machine.

**Figure 8:**
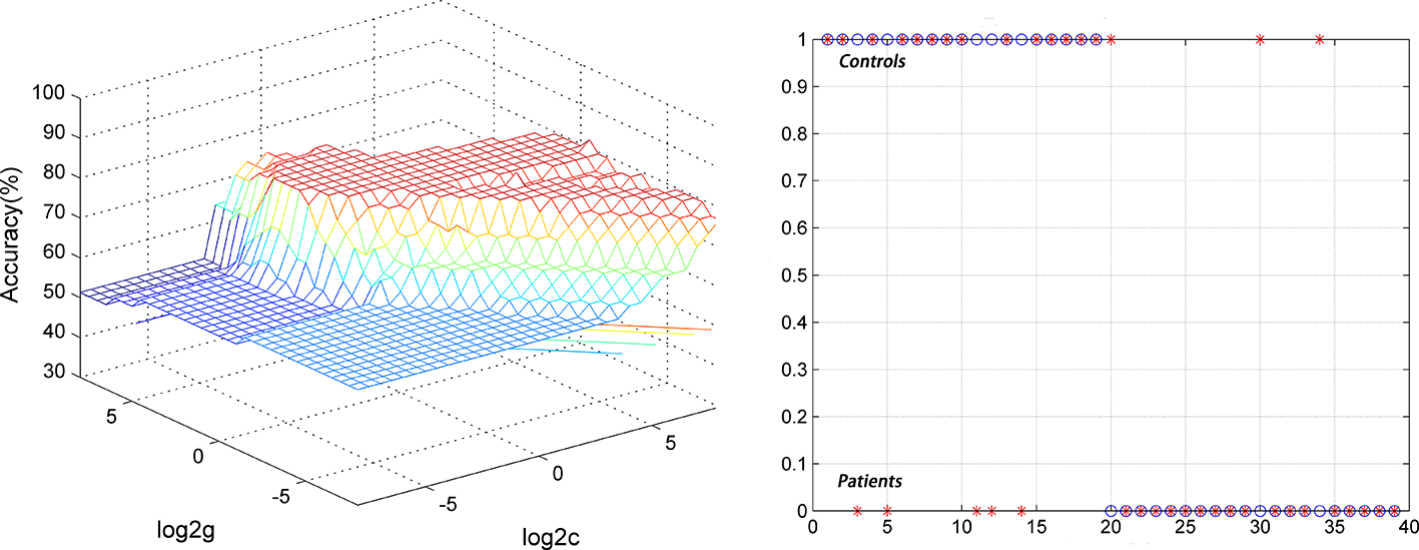
Using decreased NH values in the bilateral ventral MPFC to differentiate the patients (DPP group) from the controls. Visualization of classifications through support vector machine (SVM) using the NH values in the significantly different regions. Left: SVM parameters selection result of 3D view; Right: Classiﬁed map of the NH values in the bilateral ventral MPFC.

For the DPP group, the results showed that the NH values in the right PCC/precuneus exhibited an accuracy of 89.74%, a sensitivity of 84.21%, and a specificity of 95% to distinguish patients from healthy controls (**Figure 7**). The NH values in the bilateral ventral MPFC showed an accuracy of 79.49%, a sensitivity of 73.68%, and a specificity of 85% (**Figure 8**). The NH values in the right Cerebellum Crus II showed an accuracy of 74.36%, a sensitivity of 68.42%, and a specificity of 80%. The NH values in the bilateral superior MPFC showed an accuracy of 79.49%, a sensitivity of 68.42%, and a specificity of 90%.

For the DT group, SVM results showed the accuracy, sensitivity, and specificity for distinguishing patients from healthy controls were more than 0.7 in the bilateral ventral MPFC, right PCC/precuneus, bilateral precuneus, and bilateral superior MPFC.

### SVR Analyses

**Figure 9:**
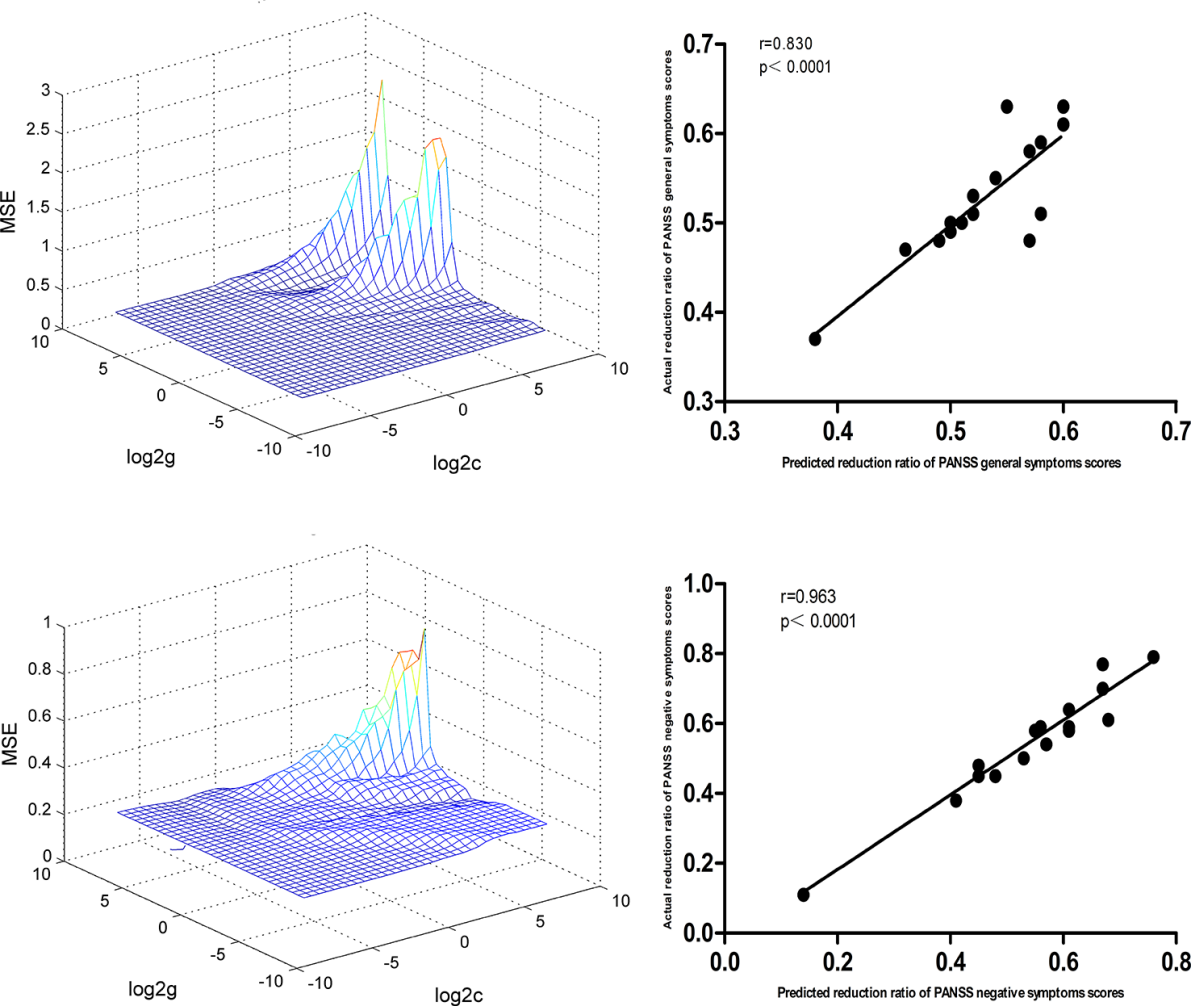
SVR results suggested that the high NH levels at baseline in the bilateral superior MPFC could predict therapeutic response in the DPP group. Left: SVR parameter selection results (3D visualization); Right: The positive correlations between predicted and actual RR of the PANSS general symptoms subscale scores (r=0.830, p < 0.0001) and negative symptoms subscale scores (r=0.963, p < 0.0001) of the individual patients after 8 weeks of DPP treatment. PANSS, Positive and Negative Syndrome Scale; RR, reduction ratio.

At p < 0.05/16 = 0.003125 level (Bonferroni correction), there were significantly positive correlations between baseline NH values in the bilateral superior MPFC and RR of the PANSS negative symptoms subscale scores (r=0.963, p < 0.0001) and general symptoms subscale scores (r=0.830, p < 0.0001) in the DPP group (**Figure 9**).

For the DT group, SVR results showed significantly positive correlations between baseline NH value in the bilateral superior MPFC and RR of PANSS total scores (r=0.887, p < 0.0001), positive symptoms subscale scores (r=0.838, p < 0.0001), and negative symptoms subscale scores (r=0.841, p < 0.0001). The details of related data are provided in the **Supplemental Figures**.

## Discussion

To our knowledge, this longitudinal study is the first to compare DMN homogeneity alterations with MCT and olanzapine therapy in schizophrenia. The results revealed that NH values in the right PCC/precuneus increased in patients in the DPP group after 8 weeks of treatment. By contrast, no signiﬁcant difference in the NH values was found in patients in the DT group from baseline to 8 weeks. Patients in the DPP group showed increased NH in the bilateral precuneus compared with patients in the DT group after 8 weeks of treatment. At baseline, no signiﬁcant difference in the NH values was found between the two groups.

Our findings show that MCT modulates DMN homogeneity in patients with schizophrenia. DPP treatment increased NH in the right PCC/precuneus, and decreased NH became almost normal after 8 weeks of treatment. By contrast, no signiﬁcant difference in the NH values was found in patients in the DT group within 8 weeks, suggesting that the effects of normalization of the DMN NH values were associated with MCT. This finding is inconsistent with the results of several previous studies, which showed that olanzapine treatment was related to the modulation of DMN connectivity (32, 33) . This inconsistency may be due to the differences of studied populations. Our study selected inpatients, and no distinction was made between first-episode and recurrent patients. The illness duration of these patients was five years or below, and the treatment period in the current study (8 weeks) was shorter than that in a previous study (33).

The precuneus and PCC play important roles in episodic memory retrieval, self-referential tasks, visuospatial imagery, and consciousness (19, 34). Previous studies have revealed that PCC is hyperactivated in patients with schizophrenia when examining themselves and others (35, 36), whereas other studies have shown decreased PCC activation in the similar conditions (37). The metabolism of the PCC region is also responsive to the cognitive state (38). Although the NH values in the right PCC/precuneus of the patients after 8 weeks of treatment were lower than those of the controls, the improvement of NH in the right PCC/precuneus might be related to the beneficial effect of MCT treatment.

DPP treatment for 8 weeks increased NH in the bilateral precuneus compared with DT treatment. At baseline, no signiﬁcant difference in the NH values was found between the two groups. Increased NH in the bilateral precuneus may reflect an enhanced interaction between the precuneus and the entire DMN. This effect may intensify the processes of self-reference and introspection in the patients. Previous study revealed that patients with schizophrenia exhibited less activation in the precuneus while working on self- and other-reﬂectivity tasks compared with controls (37). Functional neuroimaging study found that the precuneus activation was related to vividness of judgments during episodic memory retrieval (39). Accumulated evidence has revealed the important role of the precuneus in memory metacognition (40, 41). Baird et al. discovered a strong connectivity between the precuneus and anterior MPFC for memory metacognitive efficiency in resting-state functional connectivity (20). Francis et al. found that patients with early-phase psychosis who had high metacognitive capacity could bilaterally gain substantial functional connectivity of the resting state among MPFC, PCC, and precuneus (17). Our study only shows increased NH in the bilateral precuneus after DPP treatment compared with DT treatment and suggests the potential neurological mechanism of modulating DMN of MCT.

Previous studies have reported that alterations in brain function and symptomatic improvement are significantly correlated in patients with schizophrenia (42, 43). However, no significant correlation was found between altered NH and decreases in PANSS scores in the present study. Fabio et al. (32) explained that the lack of correlation might be due to the usual trajectory of reduced severity of early symptoms. After the initial treatment, the PANSS scores showed a significant downward trend in the first week of treatment. The PANSS scores declined gradually over the following weeks of treatment. Abbott et al. reported that gradual decrements in PANSS scores might mask the correlations between clinical symptoms and fMRI correlates (44).

The sensitivity or specificity of greater than 0.7 is beneficial to establish diagnostic indicators (45), whereas the sensitivity or specificity of less than 0.6 may indicate poor establishment of diagnostic indicators (46). SVM has been widely used in extensive biomedical applications for diagnosis purposes. The present SVM analyses showed that the accuracy, sensitivity, and specificity for distinguishing patients from healthy controls were more than 0.7 in the right PCC/precuneus, bilateral ventral MPFC, bilateral superior MPFC, and bilateral precuneus regions. Hence, NH in these brain regions can serve as potential image markers for distinguishing patients from controls.

Further SVR results revealed that the high NH levels at baseline in the bilateral superior MPFC could predict symptomatic improvement of PANSS after 8 weeks of DPP treatment. Previous studies revealed that the orbital frontal cortex and dorsal MPFC were related to predicting the long-term clinical outcome in the post-traumatic stress disorder (47). The hemodynamic activities of the frontotemporal cortex may predict treatment response to selective serotonin reuptake inhibitor in MDD (48). Jiang et al. found that the precuneus and left postcentral gyrus were associated with the treatment response of electroconvulsive therapy in MDD (49). Consistent with these studies, our findings that the high NH levels in the bilateral superior MPFC can predict clinical treatment response, highlight the importance of this regions in MCT and contribute to interpret clinical symptomatic improvement in psychiatric disorders.

The majority of patients with schizophrenia display neurocognitive deﬁcits. In the present study, the CF-ANF and BACS-SC scores were significantly high in the DPP group after 8 weeks of treatment relative to those in the DT group. The changes in the scores of the BACS-SC, HVLT-R, WMS-SS, and CF-ANF were also considerably higher in the DPP group than in the DT group, suggesting that MCT exerted a beneﬁcial effect on symptomatic parameters and several cognitive functions, including attention and memory. These results are consistent with several previous findings, which revealed that MCT was related to improvement in multiple neurocognitive components. Lam et al. found that MCT improved cognitive insight in patients with schizophrenia using a Chinese sample (50). Furthermore, memory delusion distress and social quality of life were improved by MCT in patients with schizophrenia, suggesting that MCT had beneﬁcial effects on symptomatic parameters and several cognitive functions (51). They also showed that patients with schizophrenia who received MCT exhibited a significant positive change in attitude toward their illness compared with the active control condition and enhanced meaning-making in patients (52). A systematic review showed that MCT was effective in reducing cognitive biases and delusions and improving insights and several aspects of neurocognitive functions in schizophrenia (9).

The present study has several limitations aside from its small sample size. First, this study focused on alterations in the DMN associated with antipsychotic and psychotherapy. Emphasizing the neurobiological contributions of the DMN at the time of therapy would be helpful. For the same reason, some meaningful changes in other brain regions may have been disregarded. Second, only 8 weeks of MCT was considered in the study. Thus, the long-term efficacy of MCT in patients with schizophrenia was not evaluated. Third, healthy controls were only scanned once at baseline. However, the data from healthy controls were used twice in the analyses. Hence, the effect of time may be not excluded in the present study. Fourth, SVM was not performed in a completely new and independent sample of patients, which might induce overoptimistic biases on the accuracies. Lastly, this study was performed at rest. The resting state of patients may be affected by symptoms. For instance, the resting state of someone experiencing auditory hallucinations is different from the resting state of someone who is inattentive. Hence, interpretation of the results must be performed with caution.

In conclusion, this present study is the ﬁrst to evaluate DMN homogeneity associated with MCT in patients with schizophrenia. MCT modulates DMN homogeneity in schizophrenia. Increased NH in the bilateral precuneus and increased NH in the right PCC/precuneus may be associated with substantial symptomatic improvement in schizophrenia induced by MCT. By contrast, no significant changes in NH are observed in patients administered with olanzapine within 8 weeks, which may be associated with poor treatment outcomes for schizophrenia. Hence, the findings contribute to the understanding of the treatment effects of MCT on brain functions in schizophrenia.

## Data Availability Statement

All datasets generated for this study are included in the article/**Supplementary Material**.

## Ethics Statement

The studies involving human participants were reviewed and approved by local ethics committee of the Second Affiliated Hospital of Xinxiang Medical University. The patients/participants provided their written informed consent to participate in this study.

## Author Contributions

All authors contributed to and approved the final manuscript. WG and YH designed the study. XS, RL, YO, and YD collected the original imaging data. WG, JZ, JC, and FL managed and analyzed the imaging data, and XS wrote the first draft of the manuscript.

## Funding

This study was supported by grants from the National Key R&D Program of China (Grant Nos. 2016YFC1307100 and 2016YFC1306900), the National Natural Science Foundation of China (Grant Nos. 81771447 and 81630033), and the Natural Science Foundation of Tianjin (Grant No. 18JCQNJC10900).

## Conflict of Interest

The authors declare that the research was conducted in the absence of any commercial or financial relationships that could be construed as a potential conflict of interest.

## References

[1] Elkis H Treatment-resistant schizophrenia. Psychiatr Clin North Am (2007) 30:511–33. 10.1016/j.psc.2007.04.001 17720034

[2] Byerly MJ Nakonezny PA Emmeline L Antipsychotic medication adherence in schizophrenia. Psychiatr Clinics North America. (2007) 30:437–52. 10.1016/j.psc.2007.04.002 17720031

[3] Voruganti LP Baker LK Awad AG New generation antipsychotic drugs and compliance behaviour. Curr Opin Psychiatry (2008) 21:133–9. 10.1097/YCO.0b013e3282f52851 18332660

[4] Erika JSI Pauliina J Noora H John JMG Sukanta S Matti I A systematic review and meta-analysis of recovery in schizophrenia. Schizophr Bull (2013) 39:1296–306. 10.1093/schbul/sbs130 PMC3796077 23172003

[5] Vohs JL Lysaker PH Francis MM Hamm J Buck KD Olesek K Metacognition, social cognition, and symptoms in patients with first episode and prolonged psychoses. Schizophr Res (2014) 153:54–9. 10.1016/j.schres.2014.01.012 24503175

[6] Masse M Lecomte T Metacognitive profiles in individuals with a first episode of psychosis and their relation to social functioning and perceived social support. Schizophr Res (2015) 166:60–4. 10.1016/j.schres.2015.05.020 26116327

[7] Macbeth A Gumley A Schwannauer M Carcione A McLeod HJ Dimaggio G Metacognition in First Episode Psychosis: Item Level Analysis of Associations with Symptoms and Engagement. Clin Psychol Psychother (2015) 23:329–39. 10.1002/cpp.1959 25963712

[8] Moritz S Woodward TS Balzan R Is metacognitive training for psychosis effective? Expert Rev Neurother (2016) 16:105–7. 10.1586/14737175.2016.1135737 26694013

[9] Pankowski D Kowalski J Gawęda Ł. The effectiveness of metacognitive training for patients with schizophrenia: a narrative systematic review of studies published between 2009 and 2015. Psychiatria Polska Press (2016) 50:787–803. 10.12740/PP/59113 27847929

[10] Briki M Monnin J Haffen E Sechter D Favrod J Netillard C Metacognitive training for schizophrenia: A multicentre randomised controlled trial. Schizophr Res (2014) 157:99–106. 10.1016/j.schres.2014.06.005 24972754

[11] Balzan RP Mattiske JK Delfabbro P Liu D Galletly C Individualized Metacognitive Training (MCT+) Reduces Delusional Symptoms in Psychosis: A Randomized Clinical Trial. Schizophr Bull (2018) 45:27–36. 10.1093/schbul/sby152 PMC6293215 30376124

[12] Moritz S Veckenstedt R Andreou C Bohn F Hottenrott B Leighton L Sustained and “sleeper” effects of group metacognitive training for schizophrenia: a randomized clinical trial. JAMA Psychiatry (2014) 71:1103–11. 10.1001/jamapsychiatry.2014.1038 25103718

[13] Philippi CL Duff MC Denburg NL Tranel D Rudrauf D Medial PFC damage abolishes the self-reference effect. J Cogn Neurosci (2012) 24:475–81. 10.1162/jocn_a_00138 PMC3297026 21942762

[14] Murray RJ Schaer M Debbané M Degrees of separation: a quantitative neuroimaging meta-analysis investigating self-specificity and shared neural activation between self- and other-reflection. Neurosci Biobehav Rev (2012) 36:1043–59. 10.1016/j.neubiorev.2011.12.013 22230705

[15] Francis MM Hummer TA Leonhardt BL Vohs JL Yung MG Mehdiyoun NF Association of medial prefrontal resting state functional connectivity and metacognitive capacity in early phase psychosis. Psychiatry Res (2017) 262:8–14. 10.1016/j.pscychresns.2016.12.014 28208070

[16] Baird B Smallwood J Gorgolewski KJ Margulies DS Medial and lateral networks in anterior prefrontal cortex support metacognitive ability for memory and perception. J Neurosci (2013) 33:16657–65. 10.1523/JNEUROSCI.0786-13.2013 PMC6618531 24133268

[17] Uddin LQ Kelly AM Biswal BB Margulies DS Shehzad Z Shaw D Network homogeneity reveals decreased integrity of default-mode network in ADHD. J Neurosci Methods (2008) 169:249–54. 10.1016/j.jneumeth.2007.11.031 18190970

[18] Guo W Yao D Jiang J Su Q Zhang Z Zhang J Abnormal default-mode network homogeneity in first-episode, drug-naive schizophrenia at rest. Prog Neuropsychopharmacol Biol Psychiatry (2014) 49:16–20. 10.1016/j.pnpbp.2013.10.021 24216538

[19] Guo W Feng L Dapeng Y Jiang J Su Q Zhang Z Decreased default-mode network homogeneity in unaffected siblings of schizophrenia patients at rest. Psychiatry Res Neuroimaging (2014) 224:218–24. 10.1016/j.pscychresns.2014.08.014 25242670

[20] Cui X Guo W Wang Y TianXiao Y XinHua Y Yefei W Aberrant default mode network homogeneity in patients with first-episode treatment-naive melancholic depression. Int J Psychophysiol (2017) 112:46–51. 10.1016/j.ijpsycho.2016.12.005 27989405

[21] Wei S Su Q Jiang M Liu F Yao D Dai Y Abnormal default-mode network homogeneity and its correlations with personality in drug-naive somatization disorder at rest. J Affect Disord (2016) 193:81–8. 10.1016/j.jad.2015.12.052 26771948

[22] Yan CG Wang XD Zuo XN Zang YF DPABI: Data Processing & Analysis for (Resting-State) Brain Imaging. Neuroinformatics (2016) 14:339–51. 10.1007/s12021-016-9299-4 27075850

[23] Hahamy A Calhoun V Pearlson G Harel M Stern N Attar F Save the global: global signal connectivity as a tool for studying clinical populations with functional magnetic resonance imaging. Brain Connect (2014) 4:395–403. 10.1089/brain.2014.0244 24923194 PMC4121047

[24] Du Y Fan Y Group information guided ICA for fMRI data analysis. Neuroimage (2013) 69:157–97. 10.1016/j.neuroimage.2012.11.008 23194820

[25] Du Y Pearlson GD Liu J Sui J Yu QB He H A group ICA based framework for evaluating resting fMRI markers when disease categories are unclear: application to schizophrenia, bipolar, and schizoaffective disorders. Neuroimage (2015) 122:S1260739814. 10.1016/j.neuroimage.2015.07.054 PMC4618037 26216278

[26] Sklar M Fast MLE Computation for the Dirichlet Multinomial. arXiv preprint arXiv: (2014) 1405:0099.

[27] Huang S Cai N Pacheco PP Narrandes S Wang Y Xu W Applications of Support Vector Machine (SVM) Learning in Cancer Genomics. Cancer Genomics Proteomics (2018) 15:41–51. 10.21873/cgp.20063 29275361 PMC5822181

[28] Chen Q Cao F Distributed support vector machine in master-slave mode. Neural Netw (2018) 101:94–100. 10.1016/j.neunet.2018.02.006 29494875

[29] Liu D Qian H Dai G Zhang Z An iterative SVM approach to feature selection and classification in high-dimensional datasets. Pattern Recognit (2013) 46:2531–7. 10.1016/j.patcog.2013.02.007

[30] Andrew AM An Introduction to Support Vector Machines and Other Kernel-based Learning Methods. Kybernetes (2000) 30:(1)103–15. 10.1108/k.2001.30.1.103.6

[31] Suykens JAK Gestel TV Brabanter JD Gestel TV Moor BD Vandewalle J Least Squares Support Vector Machines. Int J Circuit Theory Appl (2002) 27:605–15. 10.1002/(SICI)1097-007X(199911/12)27:6<605::AID-CTA86>3.0.CO;2-Z

[32] Fabio S Giuseppe B Leonardo F Grazia C Paolo T Raffaella R Treatment with olanzapine is associated with modulation of the default mode network in patients with Schizophrenia. Neuropsychopharmacology (2010) 35:904–12. 10.1038/npp.2009.192 PMC3055362 19956088

[33] Guo W Liu F Chen J Wu R Li L Zhang Z Olanzapine modulates the default-mode network homogeneity in recurrent drug-free schizophrenia at rest. Aust N Z J Psychiatry (2017) 51:1000–9. 10.1177/0004867417714952 28605934

[34] Cavanna AE Trimble MR The precuneus: a review of its functional anatomy and behavioural correlates. Brain (2006) 129:564–83. 10.1093/brain/awl004 16399806

[35] Shad MU Keshavan MS Steinberg JL Mihalakos P Thomas BP Motes MA Neurobiology of self-awareness in schizophrenia: an fMRI study. Schizophr Res (2012) 138:113–9. 10.1016/j.schres.2012.03.016 PMC3372627 22480958

[36] Holt DJ Cassidy BS Andrews-Hanna JR Lee SM Coombs G Goff DC An anterior-to-posterior shift in midline cortical activity in schizophrenia during self-reflection. Biol Psychiatry (2011) 69:415–23. 10.1016/j.biopsych.2010.10.003 PMC3740539 21144498

[37] Lisette VDM Vos AED Stiekema APM Pijnenborg GHM Tol MJv Nolen WA Insight in schizophrenia: involvement of self-reflection networks? Schizophr Bull (2013) 39:1288–95. 10.1093/schbul/sbs122 PMC3796073 23104865

[38] Robert L Sharp DJ The role of the posterior cingulate cortex in cognition and disease. BRAIN (2014) 137:12–32. 10.1093/brain/awt162 23869106 PMC3891440

[39] Richter FR Cooper RA Bays PM Simons JS Distinct neural mechanisms underlie the success, precision, and vividness of episodic memory. eLife (2016) 5:e18260. 10.7554/eLife.18260 27776631 PMC5079745

[40] Fleming SM Weil RS Zoltan N Dolan RJ Rees G Relating introspective accuracy to individual differences in brain structure. Science (2010) 329:1541–3. 10.1126/science.1191883 PMC3173849 20847276

[41] Yan MCL Brian M Janet M Ka YL Floris P Hakwan L Anatomical coupling between distinct metacognitive systems for memory and visual perception. J Neurosci (2013) 33:1897–906. 10.1523/JNEUROSCI.1890-12.2013 PMC4696871 23365229

[42] Guo W Feng L Chen J Wu R Li L Zhang Z Olanzapine modulation of long- and short-range functional connectivity in the resting brain in a sample of patients with schizophrenia. Eur Neuropsychopharmacol (2017) 27:48–58. 10.1016/j.euroneuro.2016.11.002 27887859

[43] Liu S Tao L Wei D Jiang L Wu Q Tang H Short-term effects of antipsychotic treatment on cerebral function in drug-naive first-episode schizophrenia revealed by “resting state” functional magnetic resonance imaging. Arch Gen Psychiatry (2010) 67:783. 10.1001/archgenpsychiatry.2010.84 20679586

[44] Abbott CC Jaramillo A Wilcox CE Wilcox CE Hamilton DA Antipsychotic drug effects in schizophrenia: a review of longitudinal FMRI investigations and neural interpretations. Curr Med Chem (2013) 20:428–37. 10.2174/092986713804870864 PMC3632416 23157635

[45] Swets JA Measuring the accuracy of diagnostic systems. Science (1988) 240:1285–93. 10.1126/science.3287615 3287615

[46] Gong Q Wu Q Scarpazza C Lui S Jia Z Marquand A Prognostic prediction of therapeutic response in depression using high-field MR imaging. Neuroimage (2011) 55:1497–503. 10.1016/j.neuroimage.2010.11.079 21134472

[47] Yuan M Qiu C Meng Y Ren Z Yuan C Li Y Pre-treatment Resting-State Functional MR Imaging Predicts the LongTerm Clinical Outcome After Short-Term Paroxtine Treatment in Post-traumatic Stress Disorder. Front Psychiatry (2018) 9:532. 10.3389/fpsyt.2018.00532 30425661 PMC6218594

[48] Masuda K Nakanishi M Okamoto K Kawashima C Oshita H Inoue A Different functioning of prefrontal cortex predicts treatment response after a selective serotonin reuptake inhibitor treatment in patients with major depression. J Affect Disord (2017) 214:44–52. 10.1016/j.jad.2017.02.034 28266320

[49] Jiang R Abbott CC Jiang T Du Y Espinoza R Narr KL SMRI Biomarkers Predict Electroconvulsive Treatment Outcomes: Accuracy with Independent Data Sets. Neuropsychopharmacol (2018) 43:1078–87. 10.1038/npp.2017.165 PMC5854791 28758644

[50] Lam KCK Ho CPS and Wa JC Chan SMY Yam KKN Yeung OSF Metacognitive training (MCT) for schizophrenia improves cognitive insight: A randomized controlled trial in a Chinese sample with schizophrenia spectrum disorders. Behav Res Ther (2015) 64:38–42. 10.1016/j.brat.2014.11.008 25513971

[51] Moritz S Kerstan A Veckenstedt R Randjbar S Vitzthum F Schmidt C Further evidence for the efficacy of a metacognitive group training in schizophrenia. Behav Res Ther (2011) 49:151–7. 10.1016/j.brat.2010.11.010 21276962

[52] Moritz S Mahlke CI Westermann S Ruppelt F Lysaker PH Bock T Embracing Psychosis: A Cognitive Insight Intervention Improves Personal Narratives and Meaning-Making in Patients With Schizophrenia. Schizophr Bull (2017) 44:307–16. 10.1093/schbul/sbx072 PMC5814991 29106693

