# Supplementary material for: Metacognitive Training Modulates Default-Mode Network Homogeneity During 8-Week Olanzapine Treatment in Patients With Schizophrenia
Source: Front Psychiatry. 2020 Mar 27;11:234. doi: 10.3389/fpsyt.2020.00234 (PMC7118222; doi:10.3389/fpsyt.2020.00234)
Supplement: Supplementary file 1 [file DataSheet_1.docx]

**Supplemental Methods**

**Metacognitive training (MCT)**

Each session lasted for approximately 45–60 min. In each session, patients with schizophrenia were asked to familiarize themselves with a particular bias through a comprehensive PowerPoint presentation. Then, the correlation between this particular cognitive bias and psychotic experiences was demonstrated through daily life examples, after which group exercises were executed with the objective of tackling the biases. Afterward, the patients were given assignments and leaflets with information in the form of exercises. The training program consisted of a cycle containing eight modules (free download at [www.uke.de/mct](http://www.uke.de/mct)). Module training was conducted once a week. The treatment group may have 3–10 patients. The titles of the eight modules are as follows: attribution-blaming and taking credit (emphasizing the shortcomings of monocausal inferences), jumping to conclusions I (patients are advised not to make hasty decisions), changing beliefs (teaching patients to be flexible and stay open to different interpretations), to empathize 1 (guiding patients to be aware of multiple social cues before inferring another person's mental state), memory (patients learn to refrain from strong judgments and overconfidence in false memories), to empathize II, jumping to conclusions II, self-esteem and mood [cognitive behavioral therapy(CBT)-based techniques are used to boost self-esteem]. The main purpose of MCT for patients with schizophrenia is to increase the patients’ consciousness of their cognitive distortions in an enjoyable manner and cultivate new ways of thinking and coping behavior. MCT helps alter patients’ current problem-solving repertoire.

**Default-mode network (DMN) identification**

First, subject- and group-level principal component analyses (PCAs) were performed to reduce the dimensions. The number of independent components (ICs) was assessed with the minimum description length criterion (Li et al.,2007), which was set to 20 in this study. Second, a back-reconstruction strategy was applied to acquire specific ICs based on the group ICs and PCA reduction results (Erhardt et al.,2011) . Two DMN components were selected for all participants according to the templates supplied by the GIFT (Raichle et al.,2001) . Third, a statistical map was at the threshold using voxel-wise one-sample t-tests for each component (p < 0.05 for multiple comparisons corrected by Gaussian random field [GRF] theory, voxel significance: p < 0.001; cluster significance: p < 0.05)(Song et al.,2011) . Two masks were generated. Finally, the masks were combined to form a DMN mask, which was used as a mask in the following NH analyses.

**NH analyses**

NH analyses were performed with an in-house MATLAB script. Correlation coefficients between the time series of a given voxel and the time series of all other voxels within the DMN mask were calculated for each subject. The homogeneity of a voxel was defined as the average correlation coefficient of this voxel. Then, the average correlation coefficients were transformed into z values through Fisher’s r-to-z transformation to generate NH maps. Finally the NH maps were smoothed with the Gaussian kernel of 4mm full-width at half-maximum.

**Supplemental Tables**

Table S1. The group × time interaction on DMN homogeneity

| Cluster location (group × time) | Peak coordinate | | | Cluster  (voxel) | *T* value |
| --- | --- | --- | --- | --- | --- |
|  | x | y | Z |  |  |
| Right Cerebellum Crus II | 21 | -90 | -24 | 37 | 6.4751 |
| Right Middle Temporal Gyrus | 45 | 3 | -30 | 44 | 10.096 |
| Left Middle Temporal Gyrus | -45 | 15 | -39 | 245 | 13.5894 |
| Left Anterior Cingulate Cortex | 0 | 48 | 0 | 50 | 7.4126 |
| Left Precuneus | -18 | -57 | 18 | 66 | 7.1303 |
| Right Anterior Cingulate Cortex | 9 | 42 | 21 | 46 | 8.5113 |
| Right Angular Gyrus | 51 | -57 | 30 | 111 | 9.5987 |
| Left Superior Frontal Gyrus | -6 | 36 | 39 | 93 | 21.5447 |
| Left Angular Gyrus | -51 | -66 | 39 | 121 | 10.446 |
| Right Precuneus | 3 | -45 | 39 | 231 | 10.5163 |
| Left Middle Frontal Gyrus | -39 | 18 | 48 | 35 | 5.4617 |
| Right Middle Frontal Gyrus | 27 | 36 | 48 | 67 | 9.7239 |

DMN=default-mode network

**Supplemental Figures**

**Figure S1** The brain maps of group × time interaction on NH.

NH = network homogeneity

**Figure S2** SVR results suggested that high NH levels at baseline in the bilateral superior MPFC could predict therapeutic response in the DT group. Left: SVR parameter selection results (3D visualization); Right: The positive correlations between predicted and actual RR of the PANSS negative symptoms subscale scores (r=0.841, p＜0.0001), positive symptoms subscale scores (r=0.838, p＜0.0001) and total scores (r=0.887, p＜0.0001) of individual patients after eight weeks of DT treatment.

DT=drug therapy; SVR = support vector regression; NH = network homogeneity; MPFC= medial prefrontal cortex; PANSS = Positive and Negative Syndrome Scale; RR= reduction ratio.

**Figure S1**

**
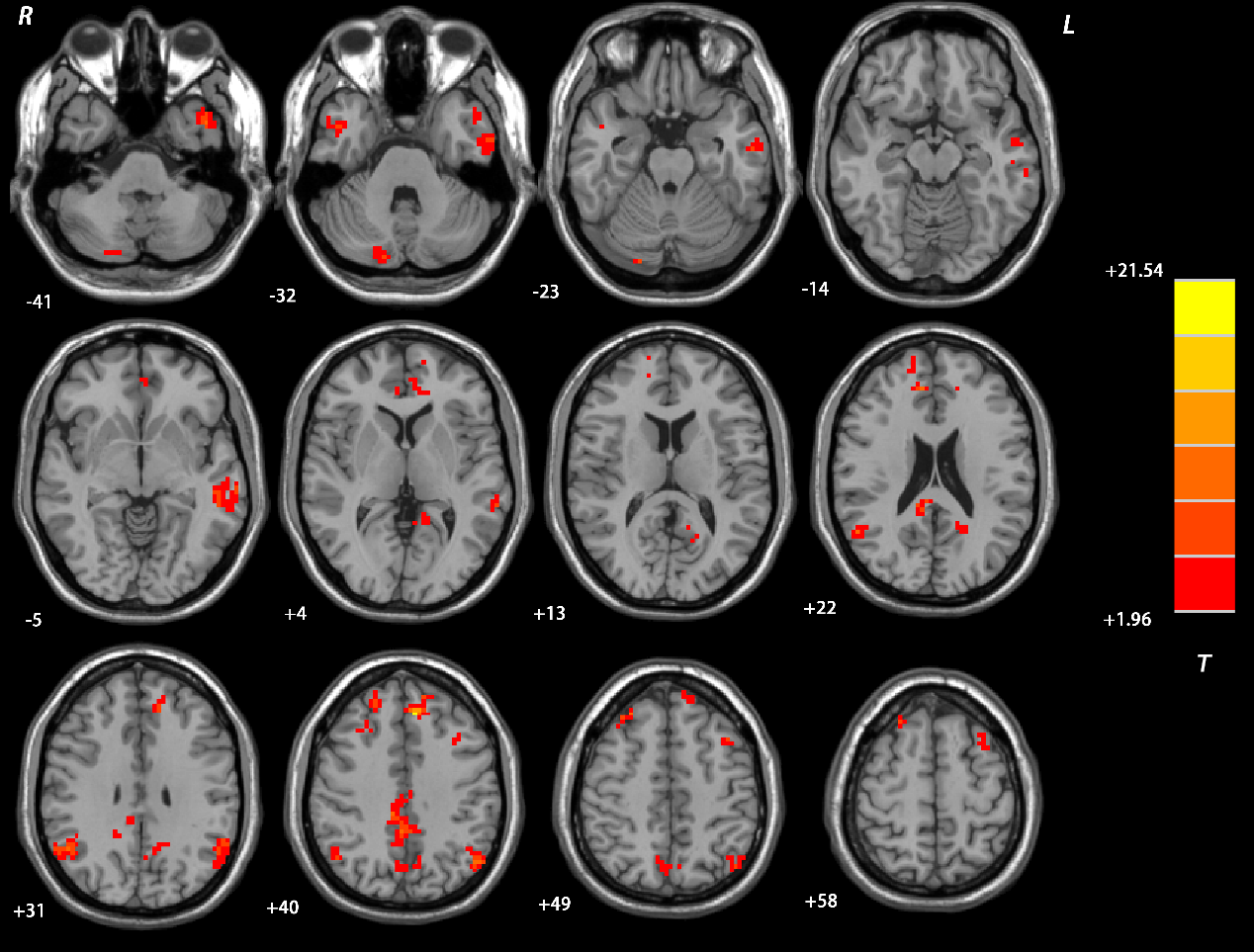
**

**Figure S2**


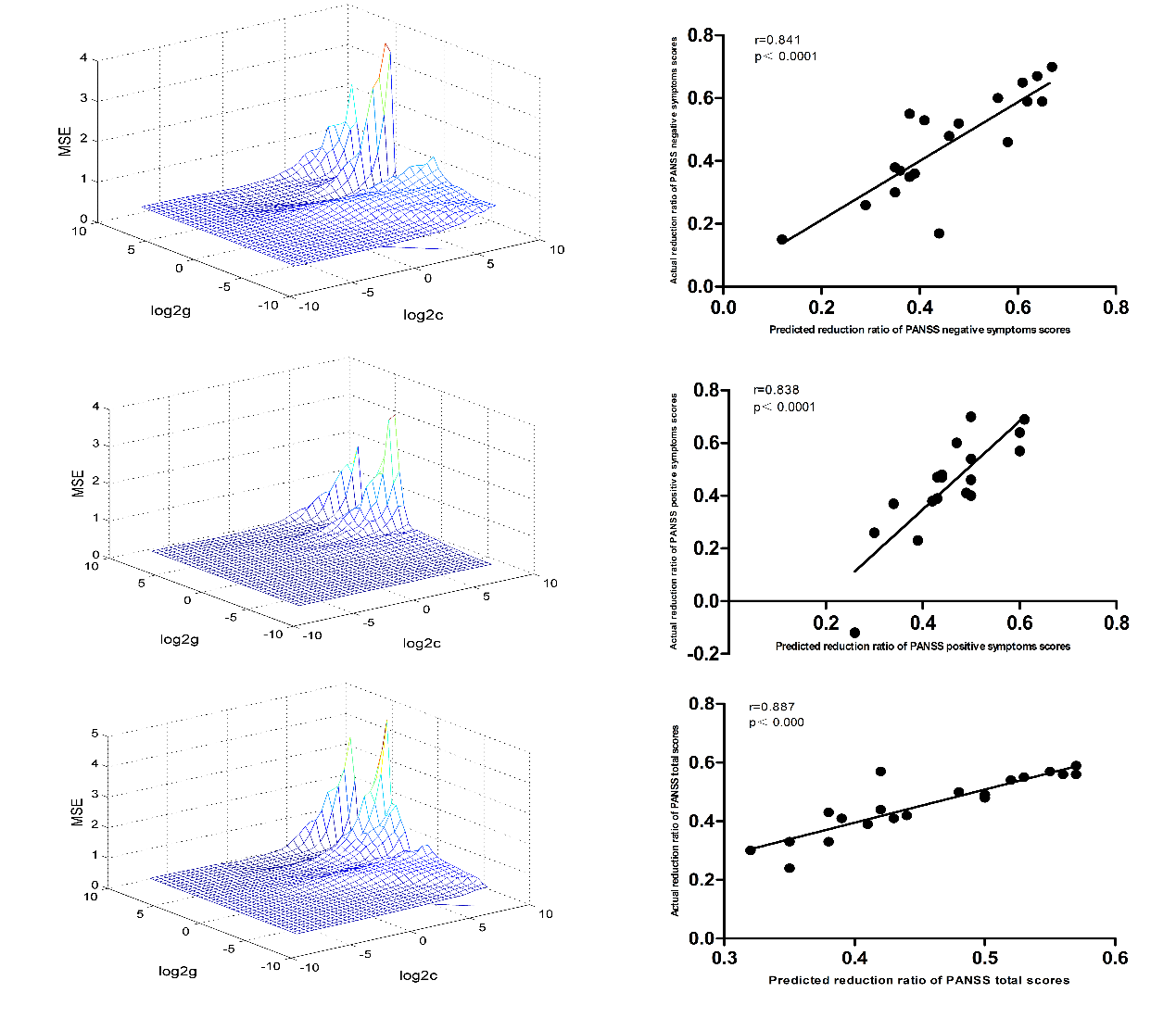


**References**

Erhardt EB, Rachakonda S and Bedrick EJ, et al. Comparison of multi-subject ICA methods for analysis of fMRI data. Human Brain Mapping .2011; 32: 2075-2095.

Hahamy A, Calhoun V, Pearlson G, et al. Save the global: global signal connectivity as a tool for studying clinical populations with functional magnetic resonance imaging. Brain Connect.2014;4: 395-403.

Li YO, Adali T and Calhoun VD. Estimating the number of independent components for functional magnetic resonance imaging data. Human brain Mapping.2007;28: 1251-1266.

Raichle ME, MacLeod AM and Snyder AZ, et al. A default mode of brain function. Proc Natl Acad Sci U S A.2001: 98: 676-682.

Song XW, Dong ZY and Long XY, et al. REST: a toolkit for resting-state functional magnetic resonance imaging data processing. PLoS One .2011;6: e25031

Yan CG, Wang XD, Zuo XN, et al. DPABI: Data Processing & Analysis for (Resting-State) Brain Imaging. *Neuroinformatics*.2016;14: 339-351.
